# Transcriptome-wide association study of inflammatory biologic age

**DOI:** 10.18632/aging.101321

**Published:** 2017-11-11

**Authors:** Honghuang Lin, Kathryn L. Lunetta, Qiang Zhao, Jian Rong, Emelia J. Benjamin, Michael M. Mendelson, Roby Joehanes, Daniel Levy, Martin G. Larson, Joanne M. Murabito

**Affiliations:** ^1^ National Heart Lung and Blood Institute's and Boston University's Framingham Heart Study, Framingham, MA 01702, USA; ^2^ Section of Computational Biomedicine, Department of Medicine, Boston University School of Medicine, Boston, MA 02118, USA; ^3^ Department of Biostatistics, Boston University School of Public Health, Boston, MA 02118, USA; ^4^ Department of Neurology, Boston University School of Medicine, Boston, MA 02118, USA; ^5^ Section of Cardiovascular Medicine and Preventive Medicine, Department of Medicine, Boston University School of Medicine, Boston, MA 02118, USA; ^6^ Department of Epidemiology, Boston University School of Public Health, Boston, MA 02118, USA; ^7^ Population Sciences Branch, National Heart, Lung, and Blood Institute, National Institutes of Health, Bethesda, MD 20892, USA; ^8^ Department of Cardiology, Boston Children's Hospital, Harvard Medical School, Boston, MA 02115, USA; ^9^ Hebrew SeniorLife, Harvard Medical School, Boston, MA 02115, USA; ^10^ Section of General Internal Medicine, Department of Medicine, Boston University School of Medicine, Boston, MA 02118, USA

**Keywords:** inflammation, gene expression, aging, epidemiology

## Abstract

Chronic low grade inflammation is a fundamental mechanism of aging. We estimated biologic age using nine biomarkers from diverse inflammatory pathways and we hypothesized that genes associated with inflammatory biological age would provide insights into human aging. In Framingham Offspring Study participants at examination 8 (2005 to 2008), we used the Klemera-Doubal method to estimate inflammatory biologic age and we computed the difference (ΔAge) between biologic age and chronologic age. Gene expression in whole blood was measured using the Affymetrix Human Exon 1.0 ST Array. We used linear mixed effect models to test associations between inflammatory ΔAge and gene expression (dependent variable) adjusting for age, sex, imputed cell counts, and technical covariates. Our study sample included 2386 participants (mean age 67±9 years, 55% women). There were 448 genes significantly were associated with inflammatory ΔAge (*P*<2.8×10^−6^), 302 genes were positively associated and 146 genes were negatively associated. Pathway analysis among the identified genes highlighted the NOD-like receptor signaling and ubiquitin mediated proteolysis pathways. In summary, we identified 448 genes that were significantly associated with inflammatory biologic age. Future functional characterization may identify molecular interventions to delay aging and prolong healthspan in older adults.

## INTRODUCTION

Non-communicable diseases remain a major contributor to morbidity and mortality in older ages. Inflammation appears to be a common pathway underlying multiple causes of death in old age [1]. Inflammatory biomarkers predict mortality [2] and age-related disease such as cardiovascular disease [2–4] as well as worsening mobility and frailty in the community [5]. Efforts to identify therapies to target chronic low grade inflammation in older adults and evaluate the impact of reduction of inflammation on important outcomes are needed [6].

Chronic low grade inflammation characterized by an imbalance of inflammatory and anti-inflammatory pathways called “inflamm-aging” is a fundamental mechanism of aging [7, 8]. Genes involved in the immune and inflammatory response pathway are associated with longevity [9] and genetic regulation of immunity is associated with human healthspan [10]. Gene expression is considered an important bridge to connect genetic variation, environmental exposures, and lifestyle factors with aging-related diseases and traits. More than one thousand genes are differentially expressed in blood in relation to chronological age, with many involved in innate and adaptive immunity, cytokine and chemokine signaling, and immune function [11].

Biological age is a measure of an individual's predicted age based on multiple biomarkers and may prove more useful in studying the biology of aging as compared to studying chronological age alone [12]. Given the importance of inflammation to aging biology, we developed a biologic age estimate based on inflammatory biomarkers representing pro-inflammatory and anti-inflammatory processes in a sample of older adults. We reported that our inflammatory biologic age measure was significantly associated with age-related morbidity and mortality [13]. The objective of the present study was to assess the association of genome-wide gene expression with inflammatory biologic age in participants from the community-based Framingham Heart Study. We hypothesized that genes associated with inflammatory biologic age would provide mechanistic insights into understanding human aging biology.

## RESULTS

Our study sample included 2386 participants (mean age 67±9 years, 55% women) from the Framingham Offspring Cohort. Descriptive characteristics of the participants are provided in Table 1.

**Table 1:** Clinical characteristics of the study sample

Characteristics	N=2386
Women, n (%)	1304 (55%)
Age, year ± SD	66.8 ± 8.9
Inflammatory BA	66.8 ±11.5
ΔAge	0.02 ±7.1
Smoker, n (%)	198 (8.3%)
Systolic blood pressure, mm Hg	129 ±17
Diastolic blood pressure, mm Hg	74± 10
Hypertension treatment	1166 (49%)
BMI kg/m^2^	28.4 ± 5.4
Total cholesterol mg/dL	186 ± 38
HDL cholesterol mg/dL	58 ± 18
Lipid treatment, n (%)	1044 (44%)
Diabetes mellitus, n (%)	406 (17%)
Cardiovascular disease, n (%)	194 (8.9%)
C-reactive protein (mg/L)	1.5 (0.8, 3.2)
Intercellular adhesion molecule 1 (ng/mL)	277 (234, 342)
Interleukin-6 (pg/mL)	1.8 (1.2, 2.9)
Lipoprotein-Associated Phospholipase A2 (Lp-PLA2) Mass (ng/mL)	202 (171, 231)
Lp-PLA2 Activity (nmol/mL/min)	137 (115, 160)
Monocyte chemoattractant protein-1 (pg/mL)	368 (302, 444)
Osteoprotegerin (pmol/L)	4.7 (3.9, 5.7)
P-Selectin (ng/mL)	40 (33, 48)
Tumor necrosis factor receptor II (pg/mL)	2383 (1940, 3050)

^+^Characteristics are represented by mean ± SD or n (%); biomarkers are median and first, third quartile

ΔAge=Inflammatory biologic age – chronologic age

### Association of inflammatory Δage with gene expression

**Figure 1:**
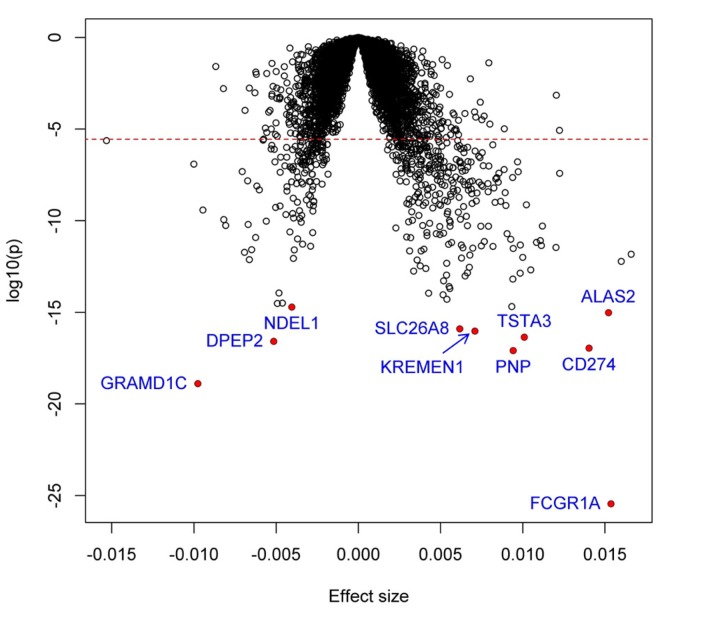
Volcano plot of association with inflammatory Δage Each dot represents one gene. The x-axis represents the beta estimation (β) of each gene, whereas the y-axis represents the log_10_(*P*). Positive effects represent that the genes were positively associated with inflammatory Δage, whereas negative effects represent that the genes were negatively associated with inflammatory Δage. The red dash line indicates *P*<0.05/17873=2.8×10^−6^.

We identified 448 genes significantly associated with the difference (ΔAge) between biologic age and chronologic age (*P*<2.8×10^−6^). Among them, 302 genes were positively associated with inflammatory Δage, whereas the remaining 146 genes were negatively associated (Supplemental Table 1). Figure 1 shows the volcano plot of all studied genes, and the top 25 associations are shown in Table 2. The most significant gene was *FCGR1A* (*P*=3.5×10^−26^), which encodes a fragment of Immunoglobulin G, known to play an important role in immune processes.

**Table 2:** Top 25 genes associated with inflammatory Δage

Affymetrix Transcript Cluster ID	Gene	Beta	SE	*P*-value
2357845	*FCGR1A*	0.0154	0.0014	3.5E-26
2636626	*GRAMD1C*	−0.0098	0.0011	1.3E-19
3527514	*PNP*	0.0094	0.0011	8.1E-18
3161082	*CD274*	0.0140	0.0016	1.1E-17
3696142	*DPEP2*	−0.0051	0.0006	2.6E-17
3157660	*TSTA3*	0.0101	0.0012	4.4E-17
3941793	*KREMEN1*	0.0071	0.0008	9.3E-17
2951730	*SLC26A8*	0.0062	0.0007	1.2E-16
4009849	*ALAS2*	0.0152	0.0019	9.6E-16
3709685	*NDEL1*	−0.0040	0.0005	1.9E-15
3061456	*SAMD9L*	0.0093	0.0012	2.1E-15
3576284	*RPS6KA5*	−0.0049	0.0006	3.0E-15
3690550	*SIAH1*	−0.0046	0.0006	3.2E-15
2421925	*GBP7*	0.0054	0.0007	5.1E-15
2700828	*SIAH2*	0.0052	0.0007	9.1E-15
2584258	*KCNH7*	0.0043	0.0005	1.1E-14
3628832	*DAPK2*	−0.0048	0.0006	1.1E-14
2828479	*SLC22A4*	0.0056	0.0007	2.0E-14
2369463	*FAM20B*	0.0055	0.0007	2.5E-14
2964200	*UBE2J1*	0.0052	0.0007	5.2E-14
3090053	*SLC25A37*	0.0094	0.0012	6.5E-14
2438531	*HDGF*	0.0065	0.0009	9.6E-14
3651955	*METTL9*	0.0054	0.0007	1.1E-13
2421883	*GBP1*	0.0098	0.0013	1.4E-13
2909404	*CD2AP*	0.0066	0.0009	1.4E-13

^+^The analyses were adjusted for age and sex

*Beta is in units of one standard deviation change in gene expression per year of inflammatory Δage; ^*^SE: standard error

We created an expression score to represent the overall association between the expression profile and inflammatory Δage using the weighted sum of all the 448 significant genes. To explore the potential utility of the expression score, we examined its association with mortality with up to ten years follow-up (mean 7.9 years, 270 deaths observed). The expression score was significantly associated with mortality after adjusting for age and sex (*P*=3.2×10^−3^, HR=1.62, 95% CI: 1.18-2.23). The association remained significant (*P*=0.048) after additionally adjusting for other mortality-related covariates, suggesting that not only the inflammatory Δage itself, but also the expression score, might be a useful biomarker to predict mortality.

### Pathway analysis

We then examined the enrichment of inflammatory Δage associated genes in biological pathways. The top 10 enriched pathways are shown in Table 3. Two pathways were significant after correction for multiple testing, including NOD-like receptor signaling pathway (*P*=2.0×10^−6^, false discovery rate=6.0×10^−4^) and ubiquitin mediated proteolysis (*P*=1.7×10^−4^, false discovery rate =0.03). The NOD-like receptor signaling pathway remained significant (*P*=4.6×10^−7^, false discovery rate =1.4×10^−4^) if we included only positively associated genes.

**Table 3:** Ten most significant canonical pathways enriched with genes associated with inflammatory Δage

KEGG pathway	#Genes in Pathway	Ratio of enrichment	*P* value	False discovery rate	Inflammatory Δage related genes in the pathway
NOD-like receptor signaling pathway	170	3.62	2.0E-06	6.0E-04	*GABARAP; GBP4; GBP5; MAPK14; CTSB; NLRP1; GABARAPL1; TAB3; GBP1; BIRC3; IFI16; IL1B; GBP7; MYD88; OAS1; STAT1; CASP5; AIM2*
Ubiquitin mediated proteolysis	137	3.25	1.7E-04	0.03	*UBE2F; UBOX5; NEDD4L; BIRC3; MDM2; UBE2J1; UBE2O; SMURF2; SIAH1; ITCH; CUL4A; UBE2L6; CDC34*
Legionellosis	55	4.35	1.0E-03	0.10	*CLK1; APAF1; IL1B; ARF1; MYD88; TLR5; CASP7*
Endocytosis	260	2.24	1.5E-03	0.11	*ARAP2; AP2M1; AP2S1; ZFYVE27; AP2A1; NEDD4L; IGF1R; IGF2R; ARF1; LDLR; MDM2; PRKCZ; SMURF2; ITCH; PIP5K1B; SNX3; DNAJC6*
Central carbon metabolism in cancer	67	3.57	3.2E-03	0.18	*AKT3; AKT1; FLT3; HK1; SLC1A5; SLC2A1; SLC7A5*
Salmonella infection	86	3.18	3.5E-03	0.18	*MAPK14; KLC3; IL1B; KLC1; MYD88; PKN2; TLR5; WASF1*
TNF signaling pathway	110	2.80	4.7E-03	0.20	*AKT3; MAPK14; AKT1; TAB3; BIRC3; IL1B; ITCH; CASP7; RPS6KA5*
Hepatitis C	133	2.57	5.4E-03	0.21	*AKT3; PSME3; MAPK14; AKT1; DDX58; EIF2AK1; LDLR; OAS1; PPP2R2D; STAT1*
Measles	136	2.52	6.3E-03	0.21	*AKT3; AKT1; DDX58; EIF2AK1; IL1B; JAK3; MYD88; OAS1; IFIH1; STAT1*
Pyrimidine metabolism	105	2.61	1.1E-02	0.32	*CMPK2; DCK; PNP; RRM2B; POLR1D; POLR2B; UPP1; ENTPD5*

### Interaction between genes associated with inflammatory Δage

**Figure 2:**
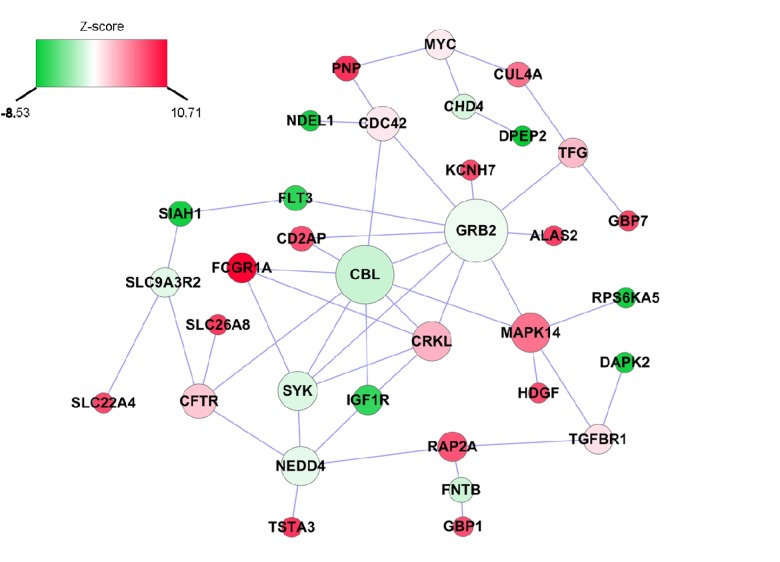
Inflammatory Δage-related subnetwork derived from protein-protein interaction Each node represents one gene, whereas each edge represents the interaction between two genes. The nodes were colored to represent their association with inflammatory Δage by z-score: red represents genes that were positively associated with inflammatory Δage, whereas green represents genes that were negatively associated with inflammatory Δage. The node size is proportional to the number of edges that the node connects to.

We also applied a dense module searching strategy [14] to identify a subnetwork containing genes associated with inflammatory Δage. As shown in Figure 2, the subnetwork is comprised of 34 nodes and 47 edges, in which each node represents one gene, and each edge represents the interaction between two genes. Two genes, *GRB2* and *CBL*, play a pivotal role in the network. *GRB2* itself was not associated with inflam-matory Δage (*P*=0.55), but it was connected with 10 other genes, of which five (*KCNH7, ALAS2, FLT3, CD2AP, MAPK14*) were associated with inflammatory Δage. Similarly, *CBL* was not associated with inflammatory Δage (*P*=0.07), but four (*FCGR1A, IGF1R, CD2AP, MAPK14*), out of its seven neighbors were associated with inflammatory Δage.

### Comparison with genes associated with chronological age

It has been long recognized that aging is an important factor affecting gene expression. An earlier meta-analysis of whole blood gene expression in 14,983 individuals reported 1,497 genes that were differentially expressed with chronological age [11]. Among them, 56 genes also were associated with inflammatory Δage, showing a significant enrichment (*P*=0.0023) by the Fisher's exact test (Supplemental Table 1), suggesting that some common mechanisms might be involved in the regulation of chronological age and inflammatory Δage.

### Enrichment of inflammatory Δage in methylation genes

**Figure 3:**
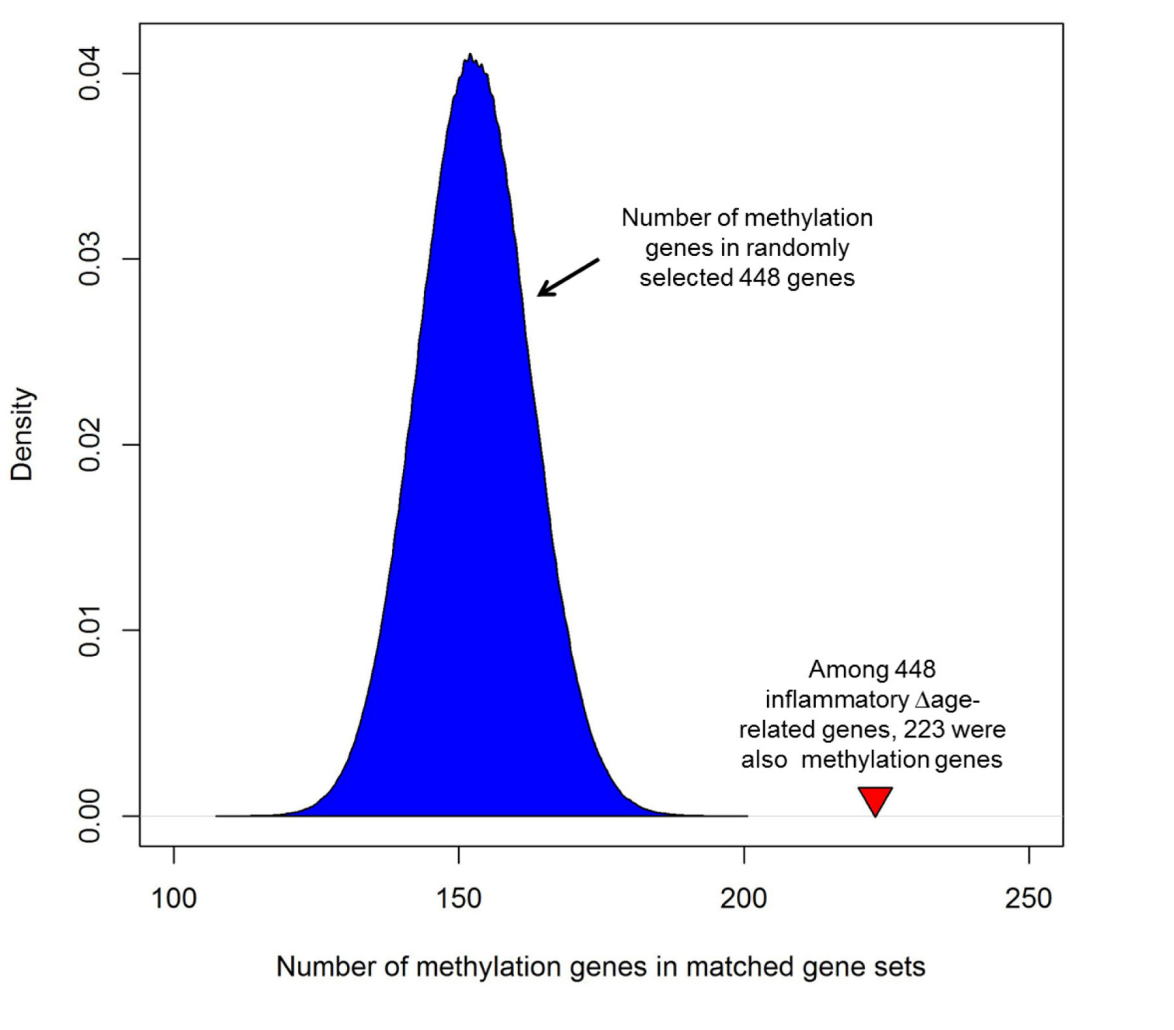
Enrichment of inflammatory Δage-related genes with corresponding differences in methylation Among 448 genes whose expression was associated with inflam-matory Δage, 223 genes contained at least one CpG where methylation was associated with inflammatory Δage (defined as differentially methylated genes [DMGs]). In order to assess its significance, we generated one million gene sets, each one containing 448 randomly selected genes and determined how many of the 448 randomly selected genes were DMGs. As shown, each randomly matched gene set contained a mean of 153 methylation genes (min: 109 genes, max: 199 genes), which is much smaller than that of inflammatory Δage-related genes (empirical p-value <1×10^−6^). The red triangleindicates the number of DMGs in inflammatory Δage-related genes.

Among 448 genes whose expression was associated with inflammatory Δage, 223 genes contained at least one CpG site in which methylation was associated with inflammatory Δage (defined as differentially methylated genes [DMGs],Supplemental Table 1). Among the 223 DMGs, 147 were positively associated with inflamma-tory Δage, and the remaining 76 genes were negatively associated with inflammatory Δage. These gene loci showed two complementary assays demonstrating altered genomic regulation in relation to inflammatory Δage. In order to assess whether this is greater than chance, we generated one million gene sets, containing 448 randomly selected genes. We then checked how many of the 448 randomly selected genes were DMGs. As shown in Figure 3 on average, each randomly matched gene set contained 153 DMGs (min: 109 genes, max: 199 genes), which is much smaller than that of inflammatory Δage-related genes (empirical p-value <1×10^−6^), indicating that there was significant overlap between genes whose expression and whose methylation were associated with inflammatory Δage. In addition, of the 56 genes associated with inflammatory Δage that were previously reported to be associated with age [11], 35 genes were DMGs (Supplemental Table 1).

We then tested the association of gene expression with inflammatory Δage by additionally adjusting for DNA methylation. Of 223 DMGs, the associations of 154 (69%) genes with inflammatory Δage were slightly attenuated after adjusting for the most significant CpG site in the gene, suggesting that DNA methylation might be a potential mechanism regulating the association between gene expression and inflammatory Δage.

## DISCUSSION

We investigated the association of gene expression with inflammatory Δage in more than 2000 older participants from the community-based Framingham Heart Study Offspring cohort. A total of 448 genes were found to be significantly associated with inflammatory Δage with two-thirds of the genes demonstrating increased expression with greater inflammatory Δage. The most significant gene was *FCGR1A*, encoding the Immunoglobulin G, which is known to activate or inhibit various cell functions, and plays a critical role in immune responses. Many inflammatory Δage-related differentially expressed genes were found to be involved in NOD-like receptor signaling pathway and ubiquitin mediated proteolysis pathway. Among the 448 genes whose expression was associated with inflam-matory Δage, 223 genes contained at least one CpG site associated with inflammatory Δage suggesting that DNA methylation may be a potential mechanism regulating the association between gene expression and inflammatory Δage.

As expected, in addition to *FCGR1A* several of the most significant genes were involved in immune and inflammatory-related functions. For example, mutations in *PNP* result in nucleoside phosphorylase deficiency that in turn produce defective cell-mediated immunity. *Pnp*-deficient mice exhibit a progressive T-cell decline with both reduced numbers of thymocytes and splenic T-cells [15]. *CD274* encodes an immune inhibitory receptor expressed on T cells, B cells and various tumor cell types. The usefulness of blocking CD274 to enhance anti-cancer immunity is under investigation [16]. Dysregulation of the immune system is one mechanism underlying inflamm-aging that accelerates biologic aging and risk for age-related disease [8].

In contrast, among the top results the *SIAH1* and *SIAH2* genes code evolutionarily conserved E3 ubiquitin ligases implicated in hypoxia and apoptosis. *SIAH1* and *SIAH2* also regulate diverse cellular functions such as DNA damage response, mitochondrial dynamics and estrogen receptor signaling [17]. These two genes have been implicated in a number of age-related diseases including Parkinson's disease and several cancers [18, 19]. A SNP in *SIAH1* was recently reported to reach a suggestive but not genome-wide level of association with exceptional longevity (living past the age that less than 1% of individuals from the 1900 birth cohort survived) [20]. In that study the longevity allele was associated with decreased risk for cardiovascular disease and hypertension. In animal models *SIAH2* appears to regulate obesity-related adipose tissue dysfunction and recruitment of immune cells to adipose tissue [21]. Adipose tissue dysfunction is an important physiologic contributor to aging related metabolic derangements, chronic disease, and frailty [22].

The NOD-like receptor signaling pathway is the biologic pathway most significantly enriched with genes associated with inflammatory Δage. In this pathway was *NLRP1* (NLR Family Pyrin Domain Containing 1), a protein coding gene that is part of the NLRP1 inflammasome initiated in response to danger signals [23]. *NLRP1* plays a role in generating innate immune responses and apoptosis. Associations with age-related diseases remain under investigation but there may be a role for susceptibility to cancer, atherosclerosis and Alzheimer disease. Other Nod-like family receptor sensors have been associated with aging and amelioration of NLRP3 mediated inflammation improves age-related declines in a number of physiologic systems [24].

DNA methylation has been recognized as an important biomarker associated with aging and age-related diseases [25–27]. Multiple computational models have been developed to predict chronological age using methylation biomarkers [28–30]. We found a significant overlap between genes whose expression was associated with inflammatory Δage, and genes that contained at least one CpG site associated with inflammatory Δage. Moreover, in more than two thirds of genes, we observed the association between gene expression and inflammatory Δage was attenuated after adjusting for the CpG site within the gene. Our results suggest that DNA methylation might be an important mechanism to regulate gene expression and thus inflammatory Δage. Both genetics and environmental factors influence gene expression and DNA methylation, which together demonstrate a dynamic landscape of chronological changes during the aging process. Future investigation of the interplay between DNA methylation and gene expression might uncover important mechanisms underlying aging, and potentially lead to better strategies to slowdown or even reverse the aging process [31, 32].

Several limitations of our study merit comment. Participants were of European descent, thus the generalizability of our findings to other race/ethnicities is unclear. Gene expression was measured from whole blood, which contains a variety of cell types and may have specific cell responses. We thereby accounted for the relative abundance of each cell type in our analyses. Our study was limited to association analyses, we cannot exclude residual confounding, and we cannot infer causality between inflammatory Δage and gene expression. In addition, the inflammatory Δage and gene expression was measured during one examination, therefore we cannot comment on longitudinal variation in the relations between gene expression and inflam-matory Δage. Our results need to be replicated in an independent sample.

However, our study also had several strengths. Our study is one of the first efforts to estimate biological age from a set of robust inflammatory biomarkers. We took a hypothesis-free approach to study transcriptome-wide profiling with inflammatory biological age in a large carefully characterized cohort. Our epigenome-wide analysis identified CpGs associated with inflammatory Δage in the many of the same genes as our transcript-tome-wide association study lending support to our findings.

In conclusion, we identified 448 genes that were significantly associated with inflammatory aging in a large community-based cohort. Future functional characterization and direct perturbation of the identified gene regulation network may enable the development of preventative strategies or therapies to arrest or slow biological aging or age-related diseases and declines of physical function.

## METHODS

### Study sample

The Framingham Heart Study is a multi-generational study initiated in 1948 to investigate cardiovascular disease and its risk factors in the community. The current study is limited to the Offspring cohort participants, who are the children of the Framingham Original cohort as well as the offspring spouses [33, 34]. They were enrolled in 1971-1975 and they have been examined every 4 to 8 years. To be eligible for this study, participants needed to attend examination 8 (2005 to 2008, n=3021) at which inflammatory biomarker and gene expression data were obtained. Participants were excluded if the following data were missing: inflammatory biomarkers (n= 279), gene expression (n= 353). The final study sample included 2386 participants. All participants provided written informed consent, and the study was approved by the Boston University Medical Center Institutional Review Board.

### Derivation of inflammatory biologic age

The inflammatory biologic age estimate comprised nine inflammatory biomarkers measured from fasting morning blood samples. Assay details have been reported and the intra- and inter- assay coefficients of variation were below 10% [35]. The inflammatory biomarkers included c-reactive protein (CRP), intercellular adhesion molecule-1 (ICAM1), interleukin-6 (IL6), lipoprotein-associated phospholipase A2 (LP-PLA2) mass, LP-PLA2 activity, monocyte chemo-attractant protein-1 (MCP1), osteoprotegerin, p-selectin, and tumor necrosis factor receptor II (TNFR2). The inflammatory biomarkers function broadly in the inflammation pathway including as acute phase re-actants, chemokines, cytokines, selectins, and cell adhesion molecules [36]. These biomarkers were modestly correlated (Pearson correlations 0.06 to 0.27 except C-reactive protein and interleukin-6, r=0.52) (Supplemental Table 2). Seven of the nine biomarkers were significantly correlated with age (Supplemental Table 2a).

We used the Klemera and Doubal method [37] to estimate inflammatory biologic age. The Klemera-Doubal algorithm includes age as one of the biomarkers and demonstrates the best performances in the precision of estimation [37] and predictive ability [12]. We defined Δage as inflammatory biologic age minus chronologic age. Thus, individuals with Δage>0 have greater biologic age than chronologic age, whereas individuals with Δage<0 have biologic age less than chronologic age. The chronologic age and Δage were not correlated (*P*>0.05) [38].

### RNA extraction and gene expression profiling

The details regarding the gene expression profiling have been described previously [39, 40]. Total RNA was isolated from whole blood using PAXgene blood tubes (PreAnalytiX, Hombrechtikon, Switzerland) and amplified using the WT-Ovation Pico RNA Amplification System (NuGEN, San Carlos, CA) according to the manufacturers' standard operating procedures. The obtained cDNA was hybridized to the Affymetrix Human Exon 1.0 ST Array (Affymetrix, Inc., Santa Clara, CA). Signal intensities from the image scanner were then quantile-normalized and log2 transformed, followed by summarization using Robust Multi-array Average [41]. The annotation for each transcript was obtained from Affymetrix NetAffx Analysis Center (version 31). Transcript clusters that were not mapped to RefSeq transcripts were excluded. A total of 17,873 distinct transcripts mapping to 17,562 unique genes were included for downstream analysis. In order to adjust for the effects of different cell counts on gene expression, cell counts were imputed using a partial least squares regression method applied to mRNA expression [42].

### Statistical analyses

Linear mixed effect models were used to test associations between inflammatory Δage and gene expression, with gene expression the dependent measure and the inflammatory Δage the exposure variable. We treated familial relatedness in the Framin-gham participants as a random variance-covariance factor. The models were additionally adjusted for sex, age, imputed cell counts, and technical covariates. All analyses were performed using the R software package (www.r-project.org), and the linear mixed effect models were implemented in the “lmekin” package. We used Bonferroni adjustment to correct for multiple testing, which was defined as 0.05/17873=2.8×10^−6^.

We also created an expression score that included genes significantly associated with inflammatory Δage. Specifically, for any participant j, the score was defined as *Score*_*j*_ = ∑_*i*_*β*_*i*_ * *G*_*i,j*_, where i represented the i^th^ significant gene, *β*_*i*_ was the beta estimate of the i^th^ gene, and *G*_*i,j*_ was the expression level of the i^th^ gene for the j^th^ participant. We then examined the association between the expression score with mortality using Cox proportional hazards regression model with clustering on pedigrees. The model was implemented in the “coxme” package, and was adjusted for age and sex. We further adjusted the model for other mortality-related covariates, including smoking, diabetes, hypertension treatment, lipid treatment, prevalent cardiovascular disease, and prevalent cancer.

### Pathway analysis

Pathway analyses were performed to identify potential biologic mechanisms among the significant genes. We used WebGestalt [43], a web-based pathway analysis tool. The enrichment was assessed by the Fisher's exact test for each KEGG pathway, and the multiple testing was accounted for by the false discovery rate approach [44].

### Construction of interaction subnetwork associated with inflammatory biologic age

In order to examine the interaction between inflammatory Δage-related genes, we constructed an interaction network using a dense module searching strategy [14]. The gene interactions were downloaded from the PINA database [45]. Before searching, each gene was assigned a score to represent its association with inflammatory Δage, equivalent to Z(p-value). Each module starts with one of the top 25 genes associated with inflammatory Δage. For each of its neighboring genes, we evaluated if adding it to the module would increase the overall module score [46], which was defined as $Z_{m}=\frac{\sum g_{i}}{\sqrt{k}}$, where k is the number of genes in the module, and *g*_*i*_ is the score of the gene i. The searching stopped if no more genes could be added. The process repeated for each of the top 25 genes, and the resulting modules were merged to build an interaction subnetwork.

### Enrichment of inflammatory Δage in methylation genes

We also examined if DNA methylation might be involved in the association between gene expression and inflammatory Δage. For each significant gene (p<2.8×10^−6^), we examined if the gene contained any CpG site that was associated with inflammatory Δage. The details of Framingham DNA methylation were described previously [47–49]. Briefly, the genomic DNA was collected from fasting peripheral whole blood, bisulfite-treated and hybridized to the Infinium HumanMethylation450 BeadChip (Illumina, San Diego, CA) according to the manufacturer's standard protocols [50]. The methylation status was represented by the β value, and the raw data were normalized and corrected for the background noise by “DASEN” R package [51]. After quality control filters, a total of 443,252 CpG sites were then tested for the association with inflammatory Δage, and the significance cutoff for the CpG site was defined as p<0.05/N, where N was the number of CpG sites within the tested genes.

## SUPPLEMENTARY MATERIAL TABLES


